# Hospital Wastewater as a Reservoir for Antibiotic Resistance Genes: A Meta-Analysis

**DOI:** 10.3389/fpubh.2020.574968

**Published:** 2020-10-28

**Authors:** Shengcen Zhang, Jiangqing Huang, Zhichang Zhao, Yingping Cao, Bin Li

**Affiliations:** ^1^Department of Clinical Laboratory, Fujian Medical University Union Hospital, Fuzhou, China; ^2^Department of Pharmacy, Fujian Medical University Union Hospital, Fuzhou, China

**Keywords:** antibiotic resistance genes (ARGs), gene abundance, influencing factors, hospital wastewater, meta-analysis

## Abstract

**Background:** The emergence and dissemination of antibiotic resistance genes (ARGs) in the environment poses a huge global health hazard. Hospital wastewater (HWW), in which a high density of antibiotic residues and antibiotic-resistant bacteria are present, may be a reservoir of ARGs dissemination into the environment. Our meta-analysis comprehensively analyzes the prevalence of ARGs in HWW, as well as the influencing factors in ARGs distribution.

**Methods:** Online databases were used to search for literature using the subject terms: “Drug Resistance” AND “Genes” AND “Hospitals” AND “Wastewater.” Two reviewers independently applied predefined criteria to assess the literature and extract data including “relative abundance of ARGs,” “title,” “authors,” “country,” “location,” “sampling year,” and “sampling seasons.” The median values and 95% confidence intervals of ARGs abundance were calculated by Wilcox.test function in R. Temporal trends, spatial differences, seasonal variations and removal efficiency of ARGs were analyzed by Pearson correlation analysis and Kruskal-Wallis H test.

**Results:** Resistance genes to carbapenems, sulfonamides, tetracyclines and mobile genetic elements were found at high relative abundance (>10^−4^ gene copies/16S rRNA gene copies) in HWW. The abundance of resistance genes to extended-spectrum β-lactams, carbapenems, sulfonamides and glycopeptide significantly decreased, while tetracycline resistance genes abundance increased from 2014 to 2018. The abundance of ARGs was significantly different by country but not by season. ARGs could not be completely removed by on-site HWW treatments and the removal efficiency varies for different ARGs.

**Conclusions:** HWW presents more types of ARGs, and their abundance is higher than those in most wastewater systems. HWW may be a reservoir of ARGs and play an important role in the dissemination of ARGs.

## Introduction

With the extensive use of antibiotics in healthcare systems, the plantation industry and the breeding industry, the increased abundance of antibiotic resistance genes (ARGs) in environment has become a serious global public health concern (1, 2). Previous studies showed that there are large quantities of residual antibiotics and bacteria in hospital wastewater (HWW), which can exert selective pressure on propagation of antibiotic-resistant bacteria (3). Therefore, HWW probably has higher risks of ARGs dissemination than other wastewater systems, such as urban sewage systems (4, 5). Furthermore, glycopeptides, carbapenems and some other antibiotics are used more frequently in hospitals than in other places. This makes the ARG profiles of HWW different from that of other wastewater systems (6). This difference further increases the risk of hospital related ARGs dissemination.

**Figure 1:**
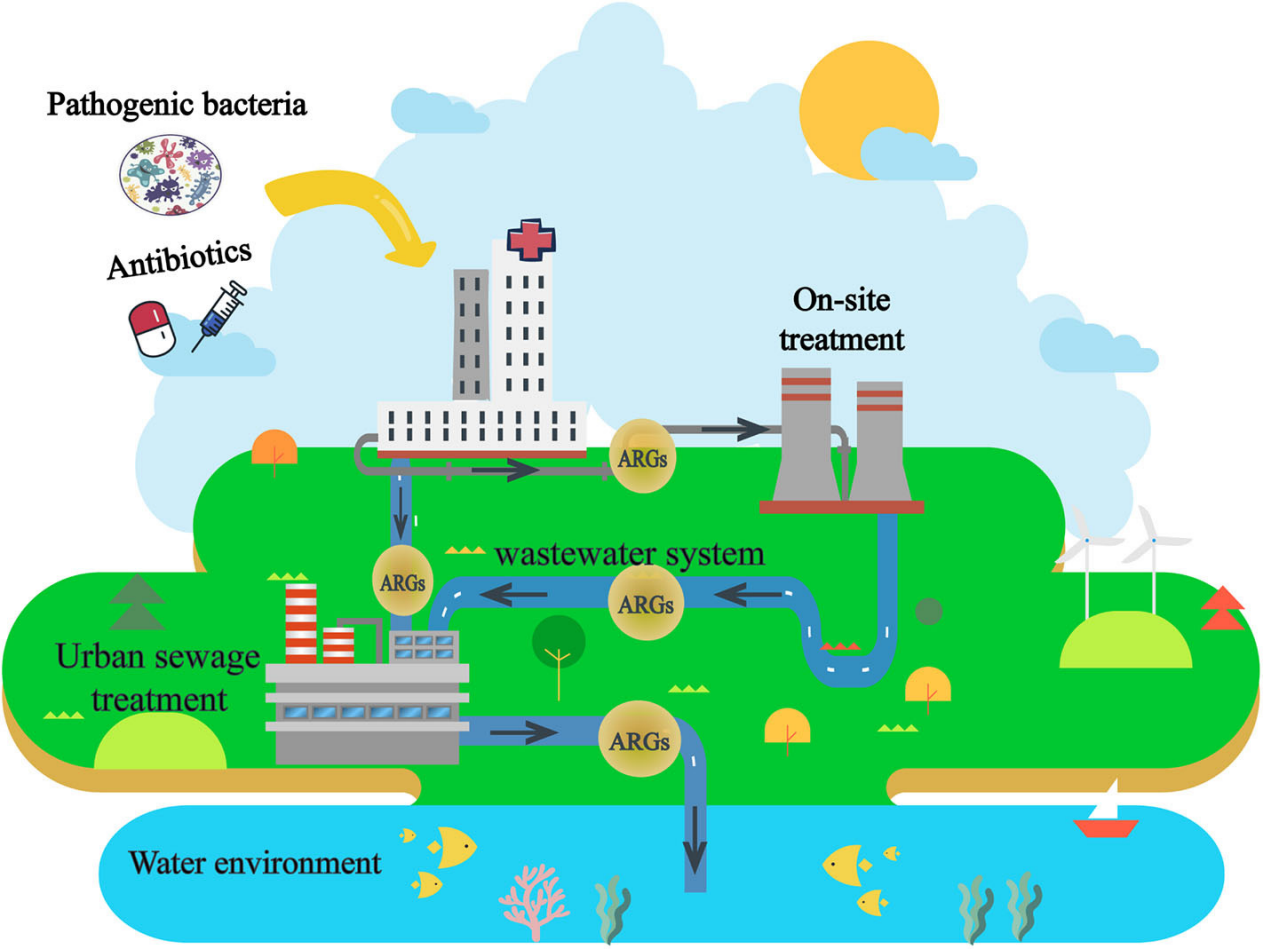
Flow of ARGs from HWW to water environment.

In order to reduce the harm of effluent after being discharged, regulations on emission standards of sludge/sewage have been established worldwide since the 1980s (7). But only a few countries (e.g., France and Italy) have established legal regulations for HWW treatment before release (8–10). Unfortunately, the biological safety assessment of ARGs is not a requested emission standard for wastewater (11). The current situation means a heavy biosafety risk of ARGs from HWW, which has been largely neglected in current waste-water treatment facilities and by associated regulations (11). Furthermore, ARGs are persistently disseminated by horizontal gene transfer within the microbial community after treatment and discharge (12, 13). Therefore, the discharge of HWW is an important factor in the dissemination of ARGs in the environment (14–16) (Figure 1).

The influence of human activities on the presence and dissemination of ARGs in the environment has attracted attention of researchers (17, 18). However, no comprehensive meta-analysis of ARGs' characteristics in HWW has been performed thus far. Our aims are to assess the prevalence of ARGs in HWW and to analyze the influencing factors of ARGs distribution in HWW, such as seasons, countries and year.

## Materials and Methods

### Search Strategy

A systematic literature search was performed on electronic databases from EMBASE, PubMed, Web of Science and MEDLINE (for articles published up until Mar 31, 2020) by two independent reviewers (SZ and JH). The titles, abstracts, and keywords were screened by using the following subject terms and connectors: “Drug Resistance” AND “Genes” AND “Hospitals” AND “Wastewater.” Research not published in English language was not considered for our meta-analysis.

### Inclusion and Exclusion Criteria

We checked the title and abstract in all studies from the databases mentioned above, and then the literature with the relevant abstracts were examined in full. The criteria for inclusion and exclusion in this study were established before two investigators (SZ and JH) reviewed the literature. In this meta-analysis, we included all cross-sectional studies that provided sufficient original data and were conducted on the prevalence of ARGs in HWW.

The exclusion criteria were as follows: short communication, meeting article, letter, conference abstract, review, case report, animal experiment or editorial. Studies using non-standard laboratory methods and culture-dependent methods, with incomplete information, were also excluded. Two reviewers (SZ and JH) independently evaluated the eligibility of identified studies.

### Quality Assessment

Two reviewers (SZ and JH) independently assessed the quality of the literature (risk of bias) using an 11-item checklist recommended by the Agency for Healthcare Research and Quality (AHRQ), and any discrepancies were resolved by consensus. This checklist is an appraisal tool designed for systematic reviews of cross-sectional studies. Some inapplicable items were omitted or modified in our study and the revised terms are as follows:

1. Define the source of information?
2. Describe in detail the information of the hospitals?
3. Indicate the time period and type for the collection of samples?
4. Does the wastewater sample come from a general hospital of a certain size (>300 beds)?
5. Indicate if evaluators of subjective components of the study were masked to other aspects of the status of the participants?
6. Describe any assessments undertaken for quality assurance purposes (e.g., test/retest of primary outcome measurements)?
7. Describe how confounding was assessed and/or controlled.

In accordance with the AHRQ guidelines, articles were assessed by each item being answered as “yes” “no,” or “unclear.” An item is scored “1” if it is answered “YES” and it indicates low risk of bias; if it is answered “NO” or “UNCLEAR,” then the item is scored “0.” Article quality is assessed as follows: low quality = 0–3; moderate quality = 4–5; high quality = 6–7.

### Data Extraction

The main outcome of the included articles is relative abundance of ARGs (obtained by normalizing their copy numbers to 16S rRNA gene copy numbers) or absolute abundance of ARGs and 16S rRNA gene (gene copies/ml). Data were also extracted for the following variables: title, author, country, location, sampling year, sampling season. When the information was incomplete, the authors were contacted to request the accurate data.

Reported abundance below the limits of detection or not detected were entered as zero values. Intended data was extracted independently from each study into pre-prepared forms by using a standardized extraction criteria and any discrepancies were resolved by consensus.

### Statistical Analyses

We assessed the prevalence of ARGs in terms of relative abundance. The median values and 95% confidence intervals for ARGs relative abundance was calculated with the Wilcox.test function in R. The temporal trends of ARGs in HWW around the world were assessed by a two-tailed Pearson correlation analysis. Spatial differences of ARGs abundance and removal efficiency of ARGs were analyzed by Kolmogorov-Smirnov test (for two groups of samples). The Kruskal-Wallis H test (for more than two groups of samples) was performed to determine the relationship between the seasons and ARGs abundance. All analyses were performed in R software (Version 3.6.2) with a significance level of α = 0.05.

## Results

### Literature Search

**Figure 2:**
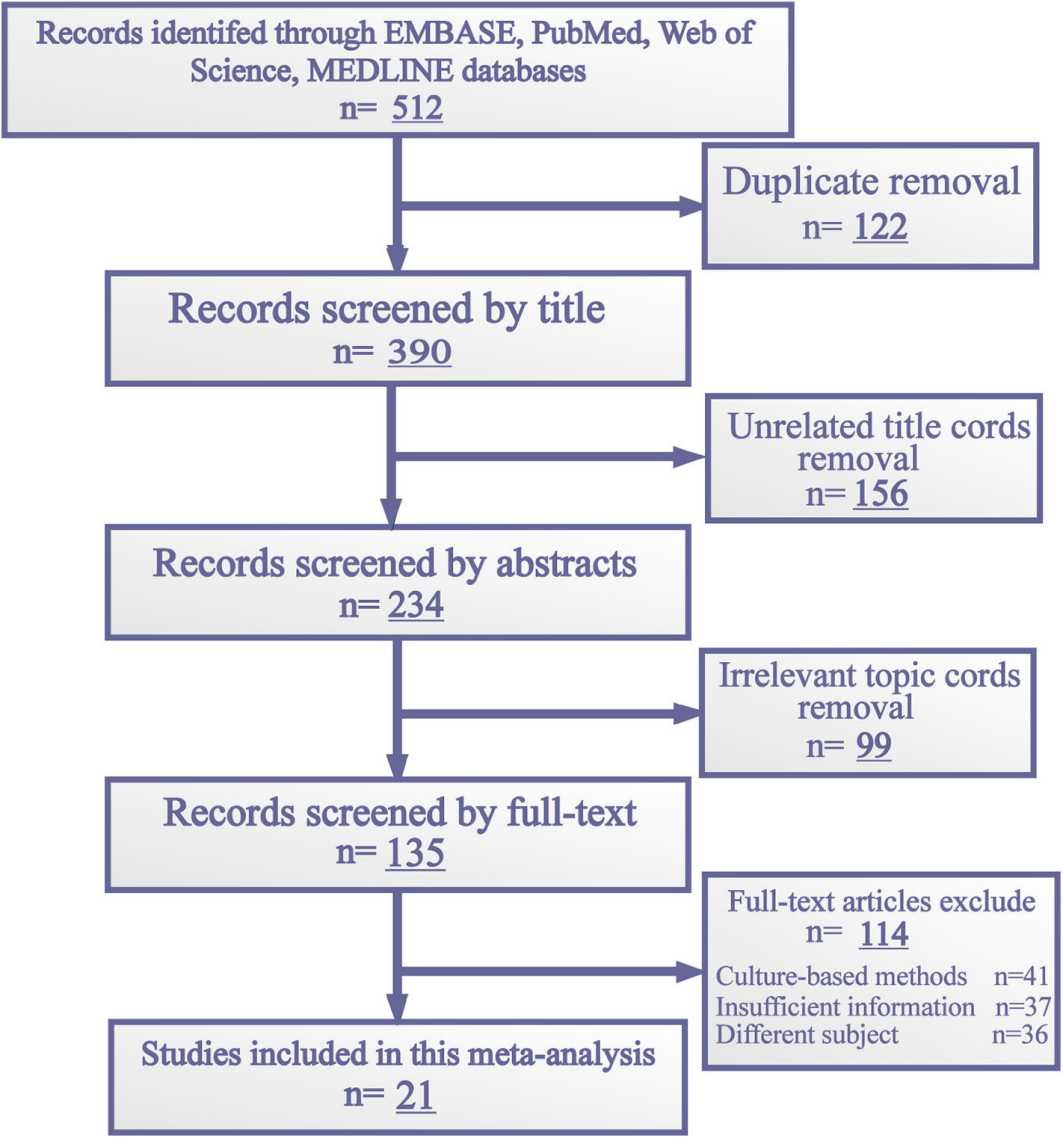
Flowchart of study selection procedure.

The initial search yielded 512 articles published up until Mar 31, 2020. All of them were found in the literature databases mentioned above. Two reviewers (SZ and JH) omitted 122 duplicate articles using a reference management tool (EndNote X9) and examined the remaining publications individually to eliminate irrelevant articles. Because of unrelated topic and non-selected type, 225 articles were eliminated. Then, 135 full texts were assessed. Of these, 41 studies were excluded for culture-based methods and 73 were excluded for incomplete data and uncorrelated subject. Finally, 21 relevant studies were selected for this meta-analysis. The flowchart for the selection of literature is shown in Figure 2.

### Study Characteristics

The included 21 studies cover different areas of the world, with the majority of studies conducted in Europe and Asia. Eleven studies were conducted in European countries (Romania, Belgium, Portugal, France, Netherlands, Switzerland, Spain, and Turkey); and eight studies were conducted in Asia (China, India, Singapore, and Saudi Arabia). The remaining two studies were conducted in Africa and America, respectively. A total of 18 ARGs and three mobile genetic elements (MGEs) were selected for analysis from these studies, including resistance genes to extended-spectrum β-lactams (*n* = 4) (13, 16, 19–25), carbapenems (*n* = 2) (13, 20, 22, 25–29), macrolide (*n* = 1) (13, 16, 18, 20, 25, 30, 31), sulfonamides (*n* = 2) (13, 16, 18, 20, 23, 24, 30–34), tetracyclines (*n* = 6) (13, 16, 18, 20, 23–25, 30–34), quinolones (*n* = 2) (13, 16, 18, 21, 24, 25, 30, 31, 33) and glycopeptide (*n* = 1) as well as MGEs (*n* = 3) (13, 18–20, 22, 25, 28, 30, 33–36). The detailed characteristics and quality assessment of the literature in this meta-analysis is shown in Table 1.

**Table 1:** Characteristics and quality assessment of included studies on ARGs in HWW in this meta-analysis.

**Authors**	**Locations**	**Years**	**Hospitals**	**Hospital types**	**Seasons**	**Treatment**	Quality assessment^*^						
							**1**	**2**	**3**	**4**	**5**	**6**	**7**
Edina Szekeres	Romania	2015	H1–H3	Oncological and General	Summer	×^c^ and **✓**^d^	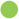	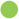	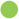	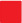	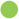	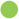	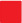
Emna Nasri	Tunis	2016	H1–H7	General	Annual average^b^	×^c^	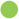	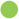	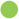	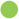	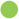	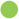	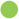
Proia Lorenzo	Belgium	2016	H1	General	Annual average^b^	×^c^	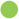	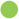	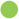	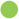	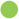	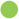	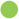
Carlos Narciso-da-Rocha	Portugal	2010	H1	General	Annual average^b^	×^c^	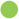	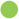	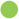	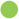	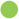	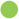	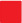
Manisha Lamba	India	2014	H1–H12	General	Summer and Winter	**✓**^d^	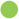	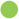	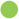	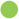	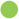	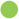	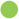
Thibault Stalder	France	2010	H1	General	Annual average	×^c^	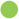	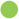	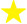	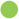	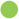	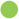	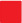
Laquaz, M.	France	2015	H1	Not mentioned	Four seasons^a^	×^c^	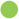	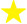	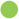	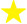	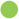	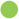	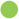
Chao Li	China	2014	H1–H5	General	Summer	**✓**^d^	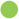	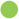	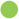	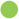	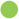	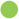	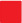
Manisha Lamba	India	2014	H1–H12	General	Summer and Winter	**✓**^d^	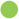	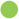	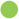	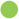	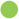	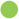	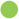
Gabriela K. Paulus	Netherlands	2017	H1–H2	Not mentioned	Spring and Winter	×^c^ and **✓**^d^	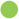	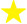	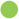	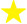	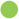	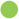	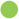
Czekalski, N	Switzerland	2011	H1	General	Spring	×^c^	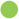	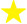	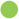	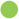	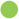	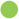	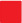
Qiang Wang	China	2016	H1–H3	General	Annual average^b^	×^c^	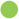	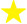	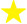	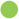	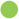	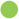	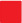
Lorenzo	Belgium	2016	H1	General	Annual average^b^	×^c^	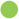	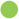	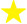	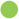	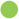	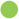	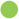
Rodriguez-Mozaz, S	Spain	2011	H1	General	Winter	×^c^	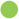	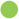	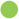	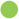	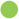	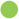	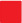
Thai-Hoang Le	Singapore	2014	H1–H2	General	Autumn	×^c^	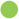	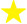	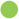	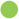	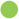	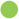	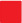
Satish Walia	USA	2015	H1	Not mentioned	Annual average^b^	×^c^	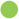	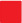	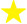	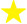	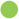	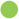	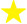
Jèssica Subirats	Spain	2016	H1–H2	Children's	Annual average^b^	×^c^	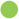	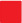	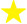	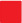	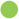	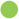	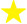
Timraz, Kenda	Saudi Arabia	2014	H1–H2	Not mentioned	Summer	×^c^ and **✓**^d^	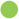	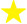	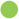	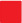	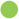	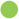	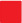
Osman Kayali	Turkey	2017	H1–H3	Not mentioned	Four seasons^a^	×^c^	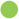	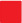	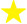	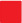	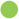	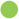	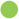
Xiaohui Liu	China	2018	H1	General	Winter	×^c^ and **✓**^d^	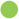	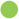	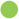	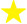	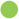	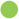	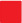
Marathe, N. P.	India	2014	H1	Not mentioned	Autumn	×^c^	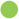	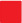	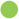	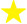	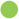	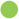	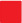

* *Green circle: the answer of this item is Yes; Red square: the answer of this item is No; Yellow star: the answer of this item is unclear or not mentioned in literature*.

a *Wastewater samples were detected in spring, summer, autumn, and winter*.

b *Included literature only provides annual averages*.

c *Literature provides the abundance of ARGs in untreated HWW*.

d *Literature provides the abundance of ARGs in treated HWW*.

### Levels of ARGs and MGEs in Effluents of Hospitals Around the World

**Figure 3:**
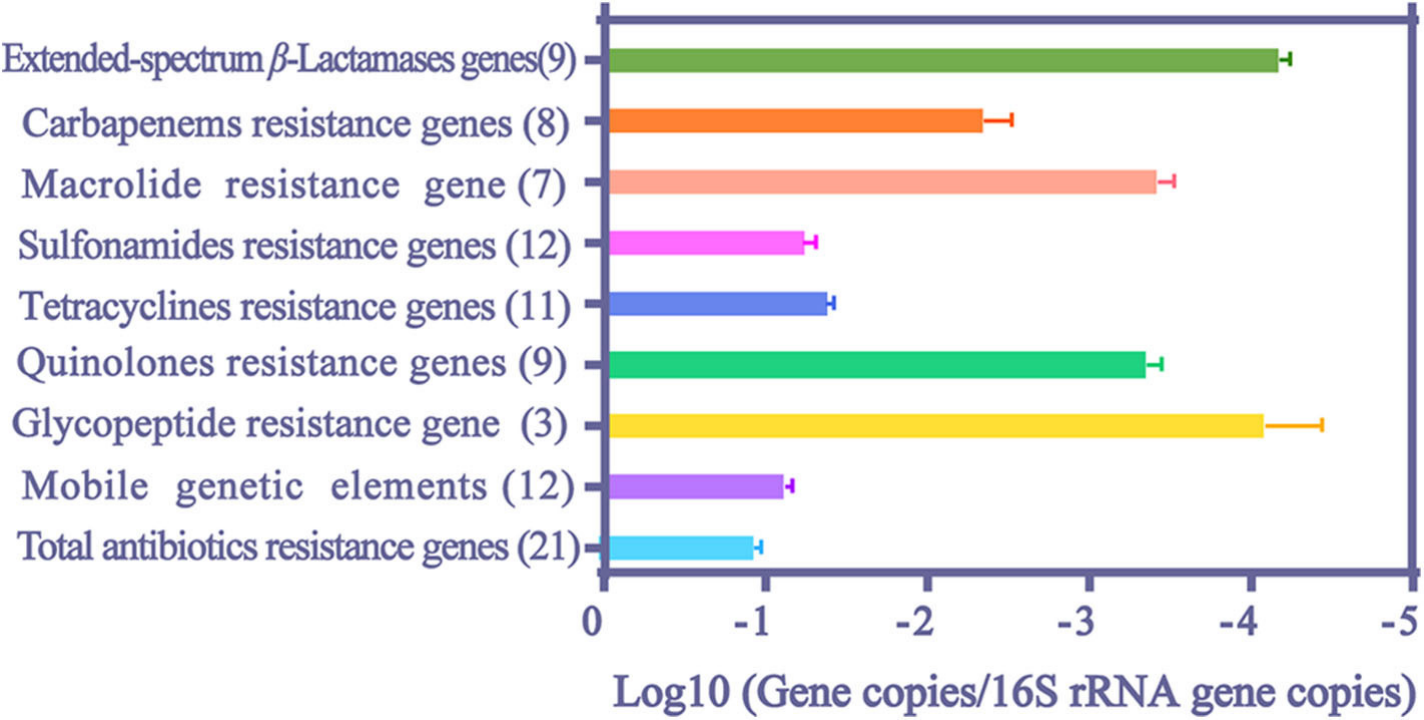
The median values and 95% confidence intervals of relative abundance of ARGs and MGEs in HWW. The number in the parentheses indicates the counts of literature.

The median values and 95% confidence intervals were calculated for the relative abundance of ARGs (Figure 3). The median values for total ARGs reached 0.111 normalized copy numbers. Among them, the values of MGEs and resistance genes to sulfonamides and tetracyclines reached a remarkably high level, ranging from 10^−2^ to 10^−1^ normalized copy numbers, and values of resistance genes to extended-spectrum β-lactams, macrolide and quinolones ranged from 10^−5^ to 10^−3^ normalized copy numbers.

Among extended-spectrum β-Lactamases (ESBLs) genes, *bla*_SHV_ and *bla*_CTX_ had the highest (8.41 × 10^−4^ normalized copy numbers) and the lowest relative abundance (1.95 × 10^−5^ normalized copy numbers), respectively (Table 2). *Bla*_NDM_ gene also reached a high value (3.98 × 10^−3^ copies/16S rRNA gene copies) and the abundance of carbapenem resistance genes (CRGs) even reached 4.55 × 10^−2^ copies/16S rRNA gene copies. The abundance of sulfonamide resistance genes ranked first among all analyzed ARGs (5.45 × 10^−2^ and 1.85 × 10^−2^ normalized copy numbers for *sul1* and *sul2*, respectively). The relative abundances of six tetracycline resistance genes were all evident in HWW (>3.42 × 10^−5^ normalized copy numbers), and the highest abundance of them was found in *tetA* (4.33 × 10^−2^ normalized copy numbers). The abundance of *vanA* and *ermB* was 8.41 × 10^−5^ and 4.76 × 10^−4^ copies/16S rRNA gene copies, respectively. *VanA* is responsible for resistance to glycopeptide and *ermB* attributes to macrolide resistance. Among all analyzed ARGs and MEGs, *intl1* showed the highest relative abundance, reaching 6.11 × 10^−2^ normalized copy numbers.

**Table 2:** Relative abundance of ARGs and MGEs in HWW (copies/16S rRNA gene copies).

**Genes**	**Gene groups**	***N*^**a**^**	**LCI**	**Median**	**UCI**
*bla_*SHV*_*	ESBL genes	4	4.52 × 10^−4^	8.41 × 10^−4^	1.93 × 10^−3^
*bla_*TEM*_*	ESBL genes	6	1.40 × 10^−5^	3.24 × 10^−4^	8.31 × 10^−4^
*bla_*CTX*_*	ESBL genes	4	1.60 × 10^−5^	1.95 × 10^−5^	2.65 × 10^−5^
*bla_*OXA*_*	ESBL genes	4	1.12 × 10^−5^	2.31 × 10^−5^	9.02 × 10^−5^
*bla_*KPC*_*	Carbapenem ARGs	6	1.64 × 10^−5^	4.37 × 10^−5^	8.32 × 10^−5^
*bla_*NDM*_*	Carbapenem ARGs	7	2.98 × 10^−3^	3.98 × 10^−3^	5.70 × 10^−3^
*ermB*	Macrolide ARG	7	3.36 × 10^−4^	4.76 × 10^−4^	5.96 × 10^−4^
*sul1*	Sulfonamide ARGs	12	4.67 × 10^−2^	5.45 × 10^−2^	6.23 × 10^−2^
*sul2*	Sulfonamide ARGs	9	1.23 × 10^−2^	1.85 × 10^−2^	2.40 × 10^−2^
*tetA*	Tetracycline ARGs	4	3.89 × 10^−2^	4.33 × 10^−2^	5.19 × 10^−2^
*tetB*	Tetracycline ARGs	4	1.94 × 10^−5^	3.42 × 10^−5^	6.49 × 10^−5^
*tetC*	Tetracycline ARGs	3	3.48 × 10^−6^	1.28 × 10^−4^	2.19 × 10^−3^
*tetO*	Tetracycline ARGs	7	4.07 × 10^−4^	4.60 × 10^−3^	1.49 × 10^−2^
*tetW*	Tetracycline ARGs	6	4.22 × 10^−3^	5.81 × 10^−3^	9.07 × 10^−3^
*tetM*	Tetracycline ARGs	6	5.19 × 10^−4^	7.91 × 10^−4^	1.11 × 10^−3^
*qnrS*	Quinolone ARGs	7	6.46 × 10^−4^	7.76 × 10^−4^	9.45 × 10^−4^
*qnrA*	Quinolone ARGs	3	5.95 × 10^−10^	4.12 × 10^−8^	2.83 × 10^−7^
*vanA*	Glycopeptide ARG	3	3.66 × 10^−5^	8.41 × 10^−5^	2.34 × 10^−4^
*intl1*	MGEs	12	5.24 × 10^−2^	6.11 × 10^−2^	6.88 × 10^−2^
*intl2*	MGEs	5	5.40 × 10^−5^	9.62 × 10^−5^	1.54 × 10^−4^
*intl3*	MGEs	6	1.79 × 10^−2^	2.90 × 10^−2^	4.11 × 10^−2^

*ARG, antibiotic resistance gene; ESBLs, extended-spectrum β-Lactamases; MGEs, mobile genetic elements; LCI, lower confidence interval; UCI, upper confidence interval*.

a *Number of studies*.

### The Temporal Trends of ARGs and MGEs in HWW

**Figure 4:**
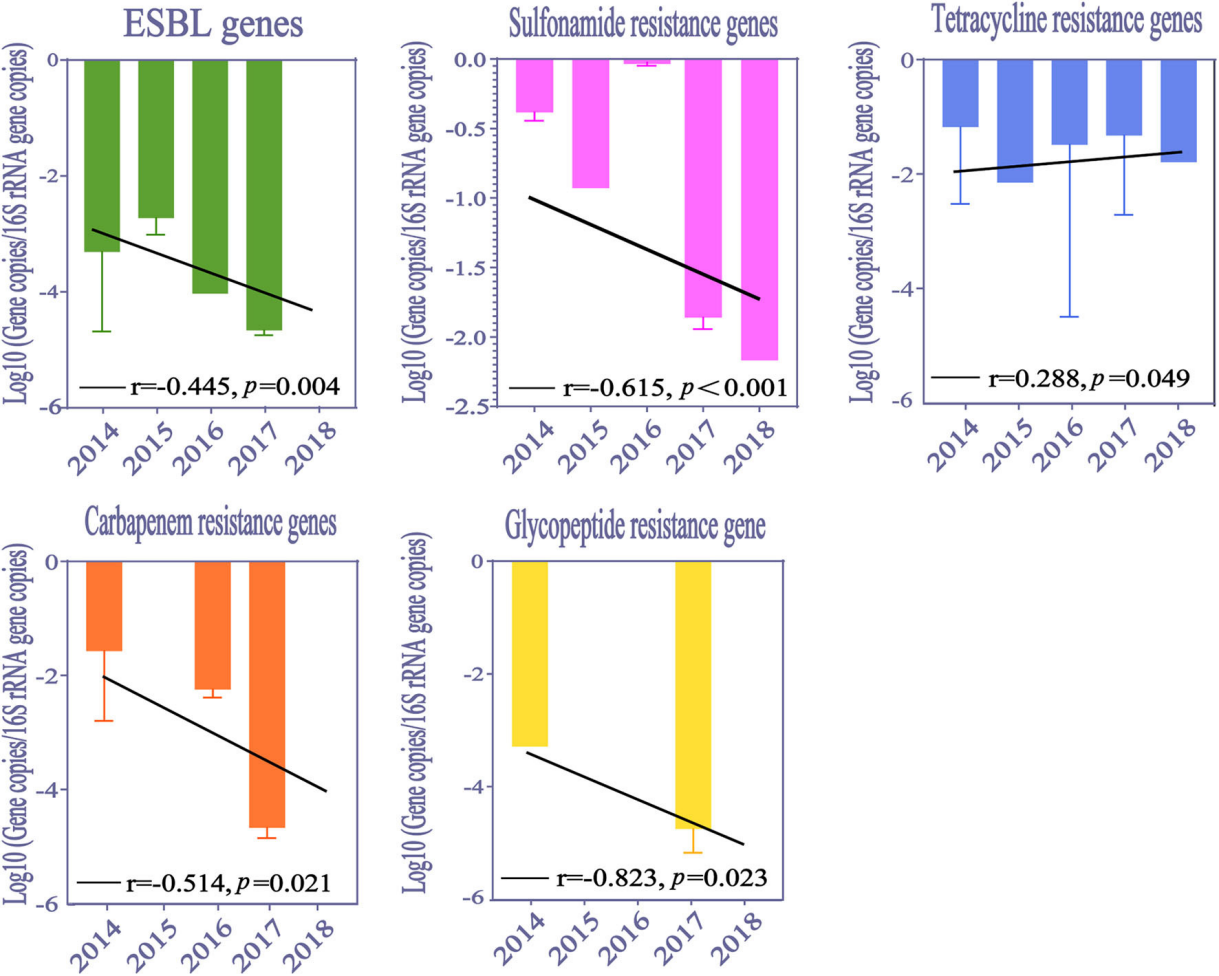
The temporal trend of ARGs in HWW around the world. ESBL, Extended-spectrum β-Lactamases.

Our study analyzed the temporal trends for ARGs abundance from 2014 to 2018 (Figure 4). The abundance of resistance genes to extended-spectrum β-lactams, carbapenems, sulfonamides, and glycopeptide significantly decreased from 2014 to 2018 (Pearson correlation analysis, *p* < 0.05). However, the abundance of tetracycline resistance genes varied across this time span. Temporal trends of macrolide resistance gene, quinolone resistance genes and MGEs are not shown in our study, which might have been caused by a deficiency of available data.

### The Spatial Differences of ARGs and MGEs in HWW

**Figure 5:**
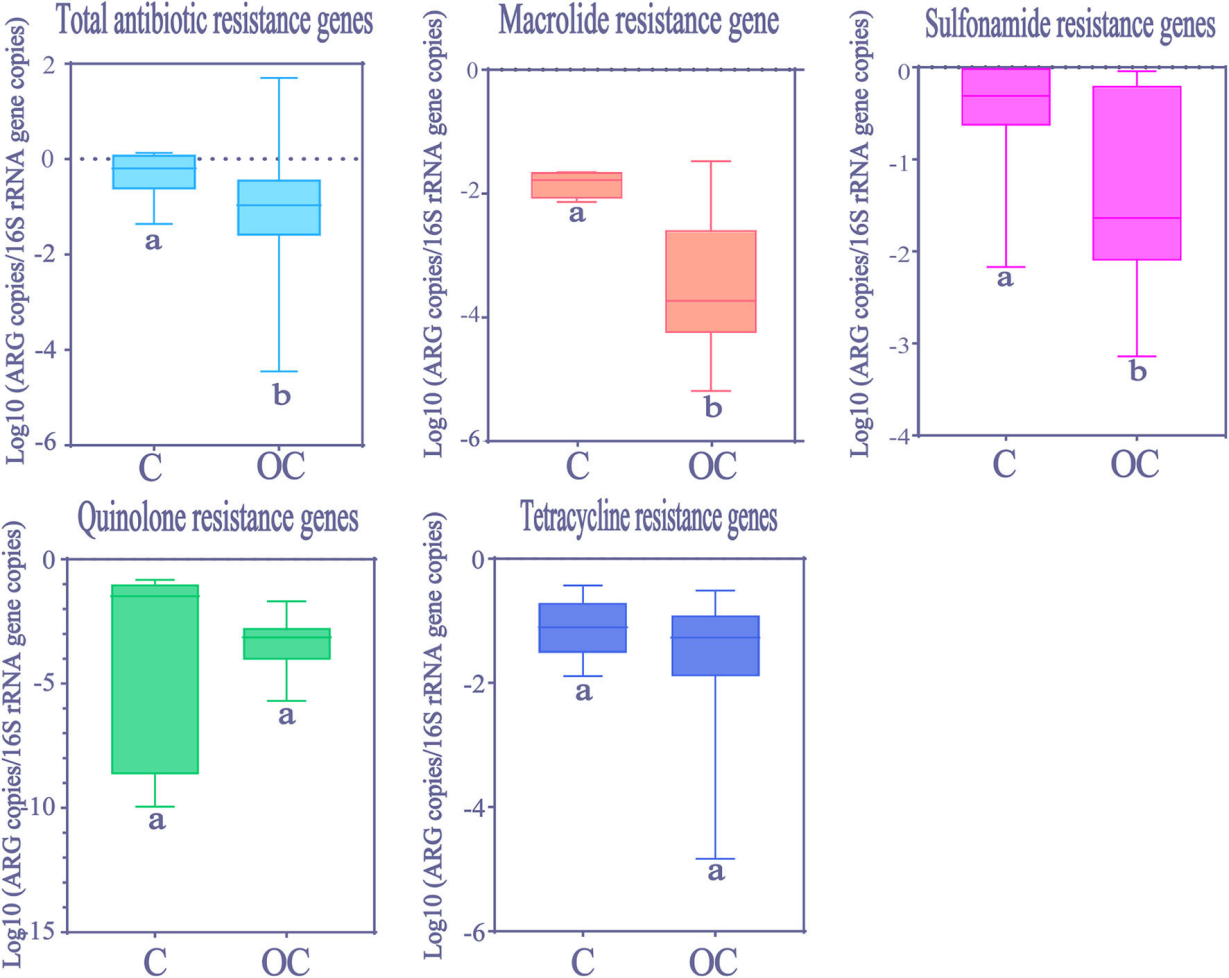
The comparison of ARGs in HWW between China and other countries around the world. Within the box plot chart, the crosspieces of each box plot represent (from top to bottom) maximum, upper-quartile, median, lower-quartile, and minimum values; different letters under the bars indicate statistically significant differences at *p* < 0.05 level. C, China; OC, Other countries.

Kolmogorov-Smirnov test showed that total ARGs abundance in Chinese HWW was significantly higher than in other countries (Figure 5). The abundance of resistance genes to macrolide and sulfonamides was higher in Chinese HWW than in other countries' HWW (Kolmogorov-Smirnov test, *p* < 0.01). The abundance of resistance genes to tetracyclines and quinolones showed no significant difference in HWW from China and from other countries (Kolmogorov-Smirnov test, *p* > 0.05).

### Seasonal Variations of ARGs and MGEs in HWW

**Figure 6:**
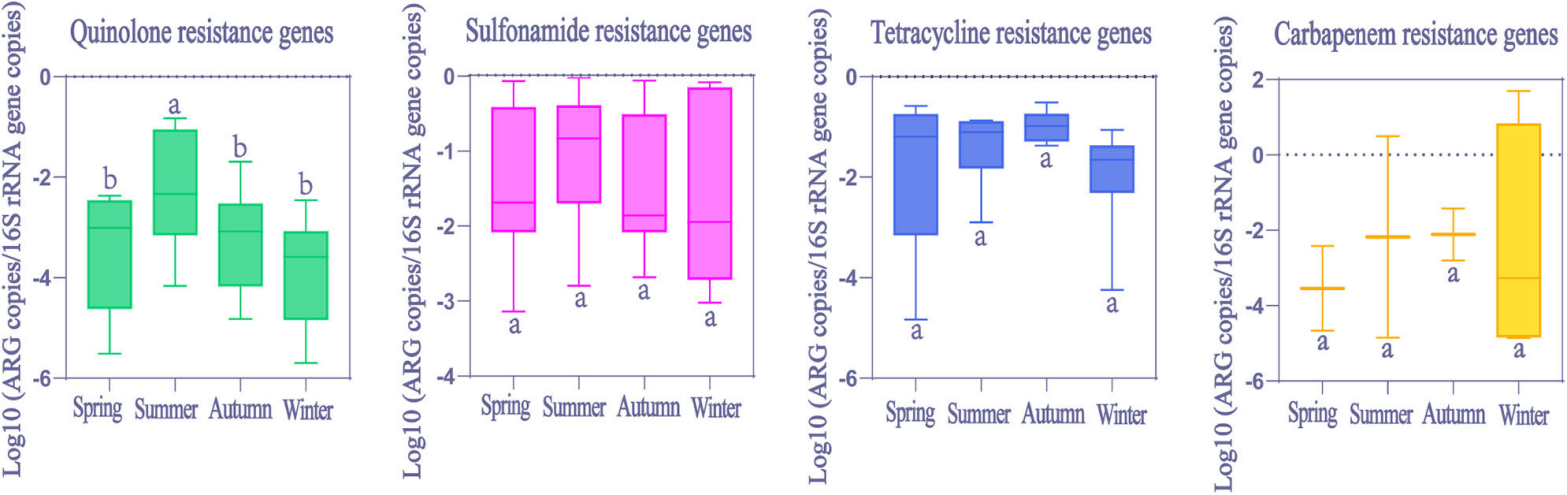
The comparison of ARGs in global HWW among different seasons. Within the box plot chart, the crosspieces of each box plot represent (from top to bottom) maximum, upper-quartile, median, lower-quartile, and minimum values; different letters under/over the bars indicate statistically significant differences at *p* < 0.05 level.

Seasonal differences in ARGs abundance were mainly not significant (Kruskal-Wallis H test, *p* > 0.05, Figure 6). However, only quinolone resistance genes showed seasonal variations (Kruskal-Wallis H test, *p* < 0.05), and the observed significant change in the relative abundance was evident only for summer.

### Removal Efficiency of ARGs and MGEs in HWW On-Site Treatment

**Figure 7:**
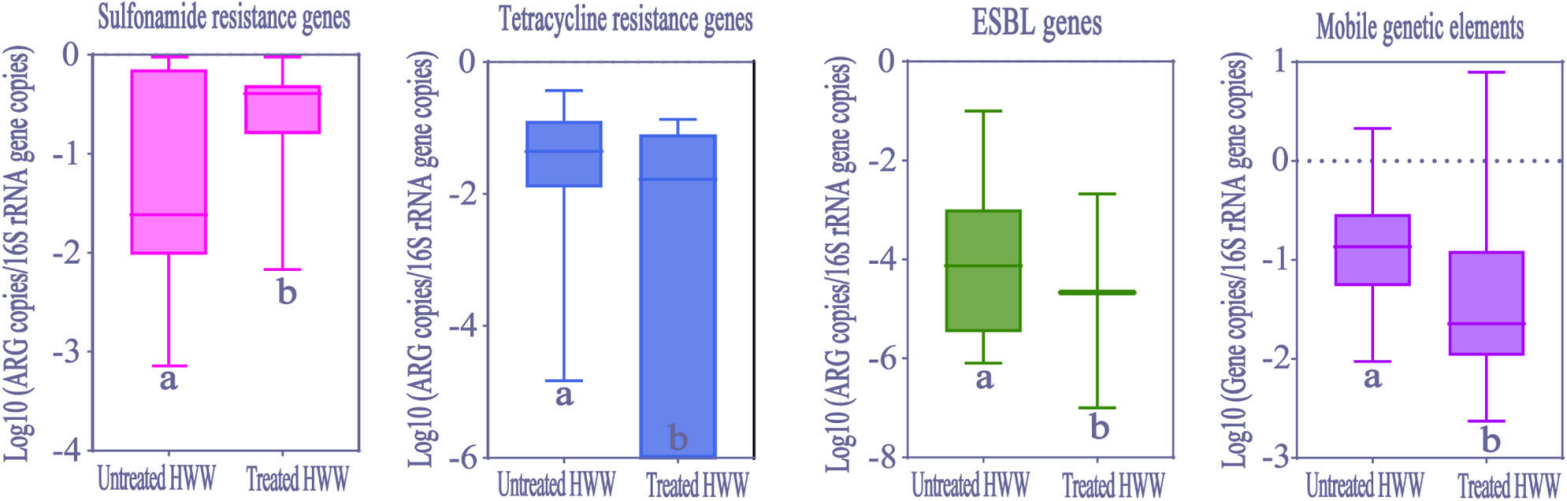
The comparison of ARGs and MGEs in treated HWW (from 37 hospitals) and untreated HWW (from 26 hospitals) around the world. Within the box plot chart, the crosspieces of each box plot represent (from top to bottom) maximum, upper-quartile, median, lower-quartile, and minimum values; different letters under the bars indicate statistically significant differences at *p* < 0.05 level. ESBL, Extended-spectrum β-Lactamases; HWW, hospital wastewater.

In order to evaluate removal efficiency of ARGs and MGEs in HWW on-site treatment, we analyzed the differences between ARGs abundance in treated wastewater from 37 hospitals (seven studies) and untreated wastewater from 26 hospitals (14 studies). Significant reduction of abundance (Kolmogorov-Smirnov test, *p* < 0.05) was observed in tetracycline resistance genes, ESBLs genes and MGEs after treatment (Figure 7). However, sulfonamide resistance genes significantly increased after treatment (Kolmogorov-Smirnov test, *p* > 0.05), which was similar to the fundings of a previous study (16). Carbapenem resistance genes abundance showed no significant difference between treated and untreated HWW.

## Discussion

The rising emergence and spread of ARGs have become a threat to human health and ecosystems (12). The contribution of HWW to the dissemination of ARGs in the environment deserves more worldwide attention (13).

In our meta-analysis, resistance genes to carbapenems, macrolide, sulfonamides, tetracyclines and quinolones were found at fairly high abundance (>10^−4^ gene copies/16S rRNA gene copies). Most of them are usually related to mobilizable and conjugative genetic elements which favor ARGs proliferation and transmission among bacterial communities (37–40). In addition, the slow flow rate of waterbody and the high amount of antibiotic residues in HWW provide an ideal environment for the enrichment of ARGs (41). Previous studies indicated that a place where the ARGs' relative abundance is higher than 10^−4^ normalized copy numbers can be considered as a highly contaminated site (42). Therefore, there is high contamination of ARGs in HWW. Furthermore, previous studies suggested that *intl1*, the main driver of ARGs dissemination in water environment, exhibits enhanced propagation ability compared with other MGEs (43, 44). The overwhelmingly high abundance of *intl1* indicates the important role of HWW in the dissemination of ARGs. The emergence of *bla*_KPC_ and *bla*_NDM_ in water environment has only been recently and rarely detected (45). However, these two ARGs reached an abundance (4.37 × 10^−5^ and 3.98 × 10^−3^ copies/16S rRNA gene copies, respectively) which could not be ignored in HWW in our study. *VanA*, uncommonly detected in environment, is usually regarded as an indicator of ARGs contamination of anthropogenic origin (26). But it also reached an abundance which could not be ignored in HWW. The emergence of glycopeptide resistance gene (8.41 × 10^−5^ copies/16S rRNA gene copies) suggests that HWW is a source of hospital-related ARGs and high value of CRGs (4.55 × 10^−3^ copies/16S rRNA gene copies) indicates the heavy contribution of HWW to the contamination of ARGs in the environment. Although one way ANOVA analysis was not performed in our meta-analysis, we found that the abundance of ARGs in HWW was higher than that in municipal wastewater systems, but no such differences were found in the comparison between HWW and pharmaceutical manufacturing wastewater or livestock wastewater (16). For example, the abundance of *ermB* in azithromycin-manufacturing wastewaters was higher than that in HWW for a high density of azithromycin residues (46–48). Meanwhile, the abundance of *sul1, sul2, tetO*, and *tetW* was higher but the abundance of *bla*_NDM_ was lower in livestock wastewater, compared with that in HWW (49, 50). This difference may be caused by extensive usage of sulfonamides and tetracyclines in graziery and higher consumption of carbapenems in hospitals. In conclusion, our study demonstrates the significant contribution of HWW to the occurrence and dispersal of ARGs in environment.

The abundance of ARGs in HWW has been recorded for more than 7 years in our included literature. Due to the limited data available, we only analyzed the temporal trends for ARGs abundance from 2014 to 2018. The overall changes in ARGs abundance presented high consistency, showing a downward trend year by year. There are two possible reasons for these results. One is that many countries have improved their regulations on antibiotics usage as antibiotic resistance has become a threat to human health. The other is that an increasing number of hospitals have performed on-site wastewater treatment to limit the diffusion of ARGs from HWW. However, the abundance of tetracycline resistance genes varied across this time span. The use of tetracyclines can be traced back to the 1940s. Long-term consumption of tetracyclines inevitably results in dissemination of tetracycline resistance genes, co-selection with other resistance genes and further causes the cross resistance of bacteria (30). Although the usages of tetracyclines have been strictly restricted in treatment of bacterial infectious diseases in recent years, they are still intensively used in stock raising and agriculture. These factors may explain why the abundance of tetracycline resistance genes was still increasing.

Due to the lack of literature on resistance genes to extended-spectrum β-lactams, carbapenems, and glycopeptide, the spatial differences of these ARGs were not analyzed in our meta-analysis. Although not always statistically significant, it does appear that the relative abundance of ARGs is higher in Chinese HWW compared with other countries (Figure 5). Given that China is a severely afflicted area in terms of ARGs pollution, the higher abundance of ARGs in Chinese HWW may not be surprising. In China, ARGs can be detected in many water environments (e.g., rivers, lakes, and groundwater), and the abundance of ARGs is higher than that in other countries (30, 51). This situation may be attributed to the huge consumption of antibiotics and the heavy burden of bacterial infectious diseases in China, as well as a lack of effective wastewater treatment equipment. Previous studies showed that China consumed 150 times more antibiotics than the UK, and the DID (daily doses antibiotics/1,000 inhabitants) in China was six times larger than that in UK (52).

In general, ARGs abundance was higher in HWW in winter due to the higher incidence rates of infectious disease and increased consumption of antibiotics in winter (22). But this is not the case in our results. No significant seasonal variation was found in ARGs abundance, except for quinolone resistance genes, whose abundance was significantly higher in summer than in other seasons. Perhaps other factors not considered in this study may be exerting stronger influences on the abundance of ARGs during different seasons, such as antibiotics (53), microbial communities (54), heavy metals (55) and economic levels. The highest abundance of quinolone resistance genes occurred in summer in our study. This may be related to warm temperatures in summer when bacteria survive longer and plasmids transform more frequently in wastewater (56). Conversely, the lower temperatures stimulate bacteria die-off in winter (28, 31, 57). Thus, further research is needed to comprehensively understand seasonal variations in ARGs abundance.

Our study shows that ARGs can only be partially removed by current wastewater treatment processes. However, more remarkably, HWW still presents high abundance of ARGs (9.11 × 10^−2^ copies/16S rRNA gene copies) after treatment. No matter in municipal sewage treatment or HWW on-site treatment, membrane bioreactor (MBR) plays an important role in removing ARGs, but its influence on ARGs removal is still limited (58). ARGs are removed by MBR treatment via size exclusion, a process of filtering out ARG-carrying bacteria (59). This treatment process may explain different removal efficiencies of various ARGs and sustained high abundance of ARGs in treated HWW. Interestingly, the increase of *sul1* abundance after treatment was observed in our meta-analysis. This may be attributed to the proliferation and spread of *sul1* among bacteria, as well as the high abundance of *intl1*, where *sul1* is usually harbored (16, 40). The selective pressure on the propagation of ARGs enhanced by the conditions generated in activated sludge is also a possible reason (60). Moreover, CRGs could not be removed by on-site treatment, contributing to the dissemination of hospital-related CRGs in water environment. Therefore, countries should pay more attention to the removal efficiency of ARGs in HWW by on-site wastewater treatment. Overall, the current HWW treatments are not the most efficient methods to remove ARGs, and it is urgent to develop more efficient wastewater treatment processes to limit the dissemination of ARGs. Our observations undoubtedly demonstrate HWW is an important reservoir and source of ARGs.

However, there are some limitations to our meta-analysis which should be considered in further research for a more comprehensive understanding of ARGs characteristics in HWW. One is that researchers tend to detect those ARGs with high detection rates in previously published studies. The other is that ARGs in treated wastewater and those in untreated wastewater are analyzed together due to the limited data available. Therefore, we clearly presented our methods and data, so that further study can compare results.

## Conclusions

Our results indicate HWW is an important reservoir of ARGs and current wastewater treatment processes are inefficient in terms of ARGs removal. Thus, in order to limit ARGs contamination from HWW to environment, further research on wastewater treatment processes that target ARGs is needed.

## Data Availability Statement

The datasets used and/or analyzed during the current study are available from the corresponding author on reasonable request.

## Author Contributions

SZ and JH performed experiments and analyzed the results. SZ conceived the study and prepared the manuscript. ZZ modified the figure. BL and YC supervised the study. All authors contributed to the article and approved the submitted version.

## Conflict of Interest

The authors declare that the research was conducted in the absence of any commercial or financial relationships that could be construed as a potential conflict of interest.
